# Regional geochemical baseline concentration of potentially toxic trace metals in the mineralized Lom Basin, East Cameroon: a tool for contamination assessment

**DOI:** 10.1186/s12932-018-0056-5

**Published:** 2018-05-02

**Authors:** Mumbfu Ernestine Mimba, Takeshi Ohba, Salomon César Nguemhe Fils, Melvin Tamnta Nforba, Nozomi Numanami, Tasin Godlove Bafon, Festus Tongwa Aka, Cheo Emmanuel Suh

**Affiliations:** 1 Department of Chemistry, School of Science, Tokai University, Hiratsuka, Kanagawa 259-1292 Japan; 2 Institute of Geological and Mining Research (IRGM), P.O Box 4110, Yaounde, Cameroon; 3 School of Geology and Mining Engineering, University of Ngaoundere, P.O Box 115, Meiganga, Cameroon; 4 Compagnie Minière du Cameroun SA, P.O. Box 35561, Yaounde, Cameroon; 5 Department of Geology, Mining and Environmental Science, University of Bamenda, P.O Box 39, Bambili, Cameroon

**Keywords:** Geochemical mapping, Background values, Stream sediments, Mineralogy, Trace metals, Cameroon

## Abstract

The distribution of trace metals in active stream sediments from the mineralized Lom Basin has been evaluated. Fifty-five bottom sediments were collected and the mineralogical composition of six pulverized samples determined by XRD. The fine fraction (< 150 µm) was subjected to total digestion (HClO_4_ + HF + HCl) and analyzed for trace metals using a combination of ICP-MS and AAS analytical methods. Results show that the mineralogy of stream sediments is dominated by quartz (39–86%), phyllosilicates (0–45%) and feldspars (0–27%). Mean concentrations of the analyzed metals are low (e.g. As = 99.40 µg/kg, Zn = 573.24 µg/kg, V = 963.14 µg/kg and Cr = 763.93 µg/kg). Iron and Mn have significant average concentrations of 28.325 and 442 mg/kg, respectively. Background and threshold values of the trace metals were computed statistically to determine geochemical anomalies of geologic or anthropogenic origin, particularly mining activity. Factor analysis, applied on normalized data, identified three associations: Ni–Cr–V–Co–As–Se–pH, Cu–Zn–Hg–Pb–Cd–Sc and Fe–Mn. The first association is controlled by source geology and the neutral pH, the second by sulphide mineralization and the last by chemical weathering of ferromagnesian minerals. Spatial analysis reveals similar distribution trends for Co–Cr–V–Ni and Cu–Zn–Pb–Sc reflecting the lithology and sulphide mineralization in the basin. Relatively high levels of As were concordant with reported gold occurrences in the area while Fe and Mn distribution are consistent with their source from the Fe-bearing metamorphic rocks. These findings provide baseline geochemical values for common and parallel geological domains in the eastern region of Cameroon. Although this study shows that the stream sediments are not polluted, the evaluation of metal composition in environmental samples from abandoned and active mine sites for comparison and environmental health risk assessment is highly recommended.

## Introduction

Geochemical mapping surveys have been conducted in different parts of the world at various scales [1–9]. Although such mapping programmes were developed primarily for geochemical prospecting [10, 11], the same principles and techniques have been expanded to encompass environmental-related issues such as land use planning, agricultural development, environmental monitoring and medical geology [12–17]. The geochemical maps resulting from such surveys show the distribution (background) of the elements analyzed. The term ‘geochemical background’ was first used in exploration geochemistry [18] and a precise definition is yet to be universally accepted [19–21]. Commonly, background is used interchangeably with baseline or threshold value and may refer to element concentrations in real sample collectives due to natural processes in pristine areas or describe anthropogenic conditions [22, 23]. Considering its spatial and temporal variability, geochemical background represents the natural concentration range of an element in a given environmental medium [24]. Geochemical surveys often target diverse sampling media including rock, soil, sediment, surface water, groundwater, rain, plant and animals, with the aim of providing basic information for policymakers and industry purposes.

Stream sediments have been extensively used as a reliable medium in geochemical mapping investigations because they provide the composite sample of the catchment area upstream of the sampling point [8, 25–30]. This mixture of sediments, rock fragments and soils act not only as an ultimate sink for trace elements derived from within the catchment but are considered as sources of metals based on changes in environmental conditions which could pose pollution problems [31, 32]. Consequently, their geochemical composition is considered to be a representative of the drainage basin geology and an effective proxy for soil and groundwater [14, 33]. Nevertheless, the spatial distribution pattern of elemental levels in sediments is characterized by a high degree of diversity. Such spatial heterogeneity is due to myriad factors including the lithology and size of the basin, weathering processes, hydrological features and land use [34]. The natural weathering of mineral deposits, as well as human activities such as small-scale mining, can result in high concentrations of trace metals in stream sediments [35–37].

Most stream sediments surveys in Cameroon have focused on mineralization and provenance (e.g. [38–43]). Besides, a national geochemical mapping is yet to be implemented in Cameroon like in many countries since it is logistically demanding and considerably expensive. However, a regional geochemical survey can effectively reveal the geochemical characteristics of the sampled medium. In addition, baseline geochemical mapping of a watershed such as the Lom Basin which includes an important mining site is crucial for future environmental assessment. This region is an important prospective area for gold with extensive research having been carried out on the secondary alluvial gold and primary gold mineralization [44–48], soil quality [49, 50] and water quality [51]. On the other hand, there have been no studies on the geochemistry of active bottom sediments for environmental purposes. Thus, the determination of geochemical baseline is fundamental to setting guidelines for environmental management. In fact, geochemical mapping incorporating stream water and stream sediment is a holistic approach to understanding the bulk chemistry and the geochemical processes occurring within this heavily mineralized basin. Accordingly, Mimba et al. [52, 53] investigated the major ion and trace metal geochemistry of stream water in the Lom Basin and reported that the streams were not contaminated in spite of the past and ongoing mining activities.

The present study, therefore, focuses on the mineralogical and geochemical features of stream sediments from the lower Lom Basin. The purpose of this study is: (a) to characterize the mineralogical and trace metal composition of streambed sediments, (b) to evaluate the level of trace element contamination in sediments in comparison to local soils and Sub-Saharan Africa soil composition (c) to identify the sources of trace elements based on spatial distribution.

### Study area

#### Regional setting, hydrology and climate

**Fig. 1:**
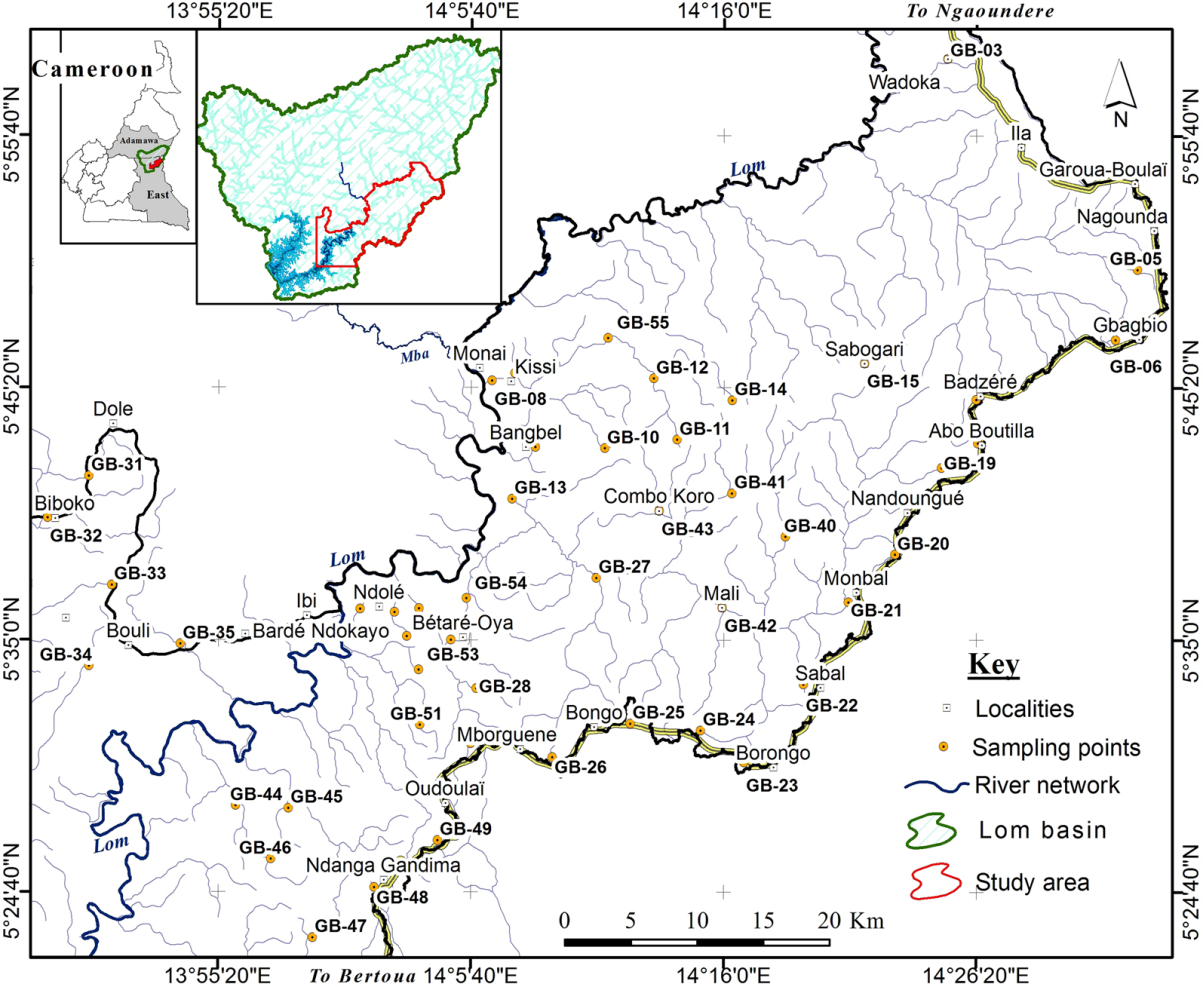
Map of study area showing the location and areas of stream sediment sample collection

A 1:50,000 scale map showing the location and sampling sites in the Lom Basin is presented in Fig. 1. The study area is found in the Lom-and-Djerem Division, East Region of Cameroon which forms part of the Equatorial Rainforest Belt. It includes two major gold districts, Betare-Oya and Garoua Boulai, and covers a surface area of 2574.67 km^2^. Monotonous, gently undulating hills of altitude ranging from 600 to 1000 m above sea level extend throughout the area. This characteristic landform has led to the development of a dendritic drainage pattern (Fig. 1). The basin is drained by the Lom River and its tributaries. Some of the lower order streams have no water flow during the dry season. During the wet period, the stream discharge increases transporting most of the stream sediments within the catchment. The study area is covered mostly by shrubs and herbaceous savanna in the north and an evergreen forest in the south. High temperatures (average, 24.7 °C), rainfall (mean annual range, 1500–2000 mm) and humidity support the luxuriant vegetation cover and enhance deep weathering of rocks and the formation of iron-rich, red ferallitic soils. There is a lack of traditional seasons but instead a long dry season from December to May, a light wet season from May to June, a short dry season from July to August and a heavy wet season from September to November.

#### Geological setting and anthropogenic activities

The Lom Basin constitutes part of the Precambrian basement of Cameroon which is divided into two significant lithostructural units; the Congo craton (CC) and the Central African Fold Belt (CAFB) [54]. The CAFB (~ 600 + 700 Ma) also known as the Pan-African belt of central Africa lies between the CC to the south and the Western Nigerian Shield to the north. This domain underlies Chad, Cameroon, Central African Republic and continues to parts of East Africa (Uganda and Sudan) [55, 56]. According to Castaing et al. [57], the evolution of this belt is due to the convergence and collision between the São Francisco–Congo cratons and the West African Craton, and a Pan-African mobile belt. In Cameroon, the present structure of the Pan-African belt can be attributed to the collision between the West Africa and Congo cratons [55].

**Fig. 2:**
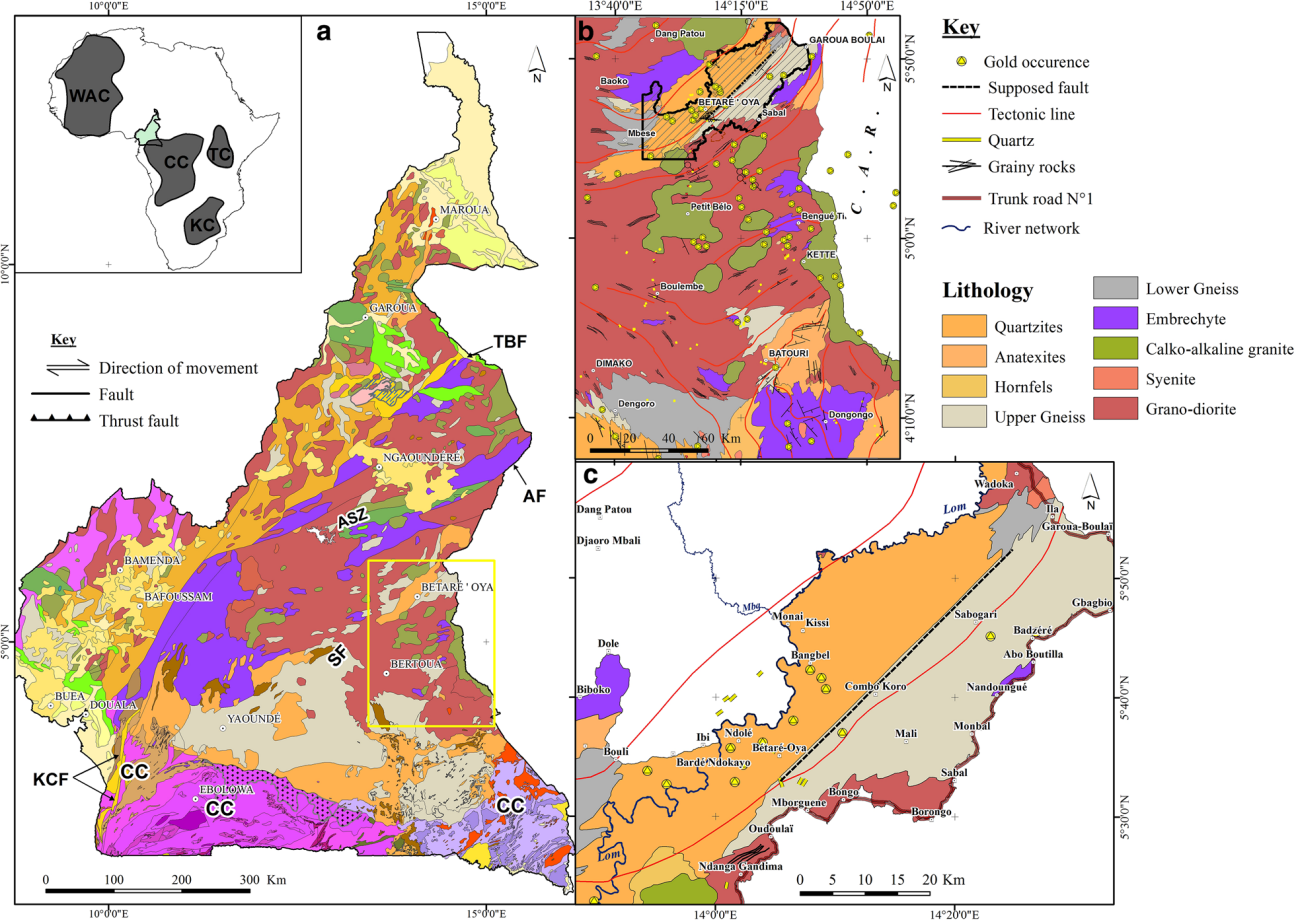
Geology of Cameroon. **a** Geological map of Cameroon (modified from Toteu et al. [95] *Faults*: Tcholliré-Banyo Fault (TBF), Adamawa Fault (AF), Sanaga Fault (SF), and Kribi-Campo Fault (KCF) *Cratons and mobile belts*: West African Craton (WAC), Tanzanian Craton (TC), Kalahari Craton (KC), Congo Craton (CC), Adamaoua Shear Zone (ASZ). **b** Regional geological map of eastern Cameroon showing reported gold indications. **c** Geology of the lower Lom Basin

Structurally, the CAFB is dominated by the Yaoundé Domain, Adamawa-Yadé Domain (AYD), and the Northwestern Cameroon Domain [55, 56, 58]. The AYD is the largest lithostructural unit of the CAFB in Cameroon. It is confined to the north by Tcholliré Banyo faults and to the south by the Yaoundé Domain (Fig. 2). This domain is marked by the development of high angle, strike-slip structures [59]. In addition, metavolcanic rocks of the Lom Basin have been identified as a major lithological group in the AYD (Fig. 2). The syn-to late-collisional calc-alkaline granitoids are ubiquitous within this domain. These rocks intrude orthogneisses representing the Paleoproterozoic basement which underwent extension and was probably dismembered during the Pan-African event. The Pan-African tectonism resulted in the formation of extensional basins including the Lom Basin [60, 61] underlain by three main units; volcaniclastic schists, metasedimentary rocks and the S-type granites [60, 62, 63]. The schists units are intercalated with quartzites and metaconglomerates and intruded by granitic plutons. The reworking of the Precambrian basement accounts for mineralization (especially gold) in the study area [55]. Consequently, alluvial gold is mined from the Lom River and its tributaries. During the last four decades, there has been a mining boom (exploration and exploitation) in the East Region of Cameroon, particularly in the districts of Betare-Oya, Batouri, Garoua-Boulai, Colomines and Kette [64, 65]. In this region, gold mining is artisanal and semi-mechanized. Gold is recovered from open pits, streambed sediments and weathered primary deposits (quartz veins) using primitive methods. Semi-mechanized exploitation often involves the use of heavy machinery. Stream sediments of the upper Lom Basin have Au concentrations of up to 450 ppm [66]. Besides small-scale mining, subsistence farming is a major form of livelihood amidst logging and timber.

## Materials and methods

### Sampling and sample preparation

Stream sediments were sampled from lower order streams draining the southeastern part of the Lom Basin, at a density of one sample every 5–10 km (Fig. 1). Sampling was done preferably near stream confluences in order to cover the whole drainage network within the area. During sampling, care was taken to avoid areas of anthropogenic influences. Nonetheless, field observations such as potential sources of contamination, land use, and upstream geology were recorded. A total of 55 active stream sediment samples were collected based on the procedures from Salminen et al. [67]. About 3 kg (to ensure that sufficient fine-grained material would be available for analysis) of the upper layer (0–10 cm) of the stream sediment was collected using a hand trowel. The wet sediment was passed through first, a 300-µm, then 150-µm stainless steel sieves set to obtain the < 150-µm fraction. This retained portion was left to settle out and excess water carefully decanted. The drained < 150-µm fraction of stream sediment was placed in clean pre-labeled polyethylene bags and air dried. Duplicate samples were collected for each site.

### Chemical analyses

In the laboratory, stream sediment samples were rinsed with Milli Q by shaking in an ultrasonic bath for 30 min each. They were then air dried and homogenized. Wet digestion and dissolution protocol (modified from Makishima and Nakamura [68]) for trace metals in the stream sediment samples was as follows. 0.15 mL of concentrated perchloric acid (60 wt% HClO_4_) and 0.3 mL of concentrated hydrofluoric acid (60 wt% HF) were added to 0.02 g of the sediment powder in a Teflon plastic bottle. The bottles were tightly capped and agitated in an ultrasonic cleanser for several hours to enhance the dissolution of samples. After complete decomposition, the bottles were uncorked, loaded on a ceramic hot plate and the samples were step-wise dried at 120, 170 and 200 °C, for 6 h at each step. Heating was carried out in a closed system. 0.2 mL of concentrated hydrochloric acid (35–37 wt% HCl) was then added and the bottle was agitated for 2 h to dissolve the degraded sample completely. Finally, the samples were dried at a temperature of 120 °C for 6 h to prevent the formation of iron oxides or hydroxides upon the final addition of nitric acid (HNO_3_). The samples were dissolved in 25 mL of 0.5 M HNO_3_ and stored in 50 mL polyethylene bottles for trace metal analysis.

Concentrations of Sc, V, Cr, Co, Ni, Cu, Zn, As, Se, Cd, Hg, and Pb in the sediments were determined using inductively coupled plasma mass spectrometry (ICP-MS) (ThermoScientific), Fe and Mn by atomic absorption spectroscopy (AAS) (contrrAA700) at the Laboratory of Volcanology and Geochemistry in Tokai University, Japan. The geochemical reference samples JA-3, JB-3 and JG-3 (Geological Survey of Japan) were used as standards. Internal standards and blanks were run at regular intervals in the analysis for quality control.

The pH of the stream sediments was measured following the procedure by the International Soil Reference and Information Center (ISRIC) [69]. A 1:2.5 ratio of solid to liquid was used. About 2 g of sediment powder was mixed with 5 mL of Milli Q in Teflon plastic bottles. The bottles were capped and agitated in an ultrasonic bath for 2 h. Prior to pH measurement, the mixture was shaken by hand and the pH of the supernatant suspension was read using a pH meter (LAQUAtwin), previously calibrated with buffer solutions of pH 4 and pH 7.

### X-ray diffraction (XRD) analysis

The relative abundances of the main silicate and oxide minerals were determined semi-quantitatively on the bulk powder using a D8 ADVANCE TKK Diffractometer with automated divergence slit and monochromatic Cu-Kα radiation (4 kV–20 mV) at the Laboratory of Inorganic Chemistry and Material Science in Tokai University, Japan. Powders from 6 pulverised (< 150 µm) representative bottom sediment sub-samples were mounted with a random orientation on an aluminum sample holder. The powder was smoothened using a slide to obtain a uniform level suitable to the X-ray beam. Each sample was scanned from 10° to 80° 2θ with a 0.5 s step. The software BRUKER-binary V4 (.RAW) was used to provide a semi-quantitative estimation of mineral content based on the diffraction patterns.

### Data processing

#### Statistical analyses

Statistical analyses were performed using Microsoft Excel and SPSS 20.0 for Windows. The statistical parameters minimum, median, mean and maximum measured the central tendency; while median absolute deviation (MAD), standard deviation, variance and coefficient of variance examined the statistical dispersion. Also, the dataset was tested for asymmetry using skewness. Univariate summary statistics showed that all measured elements were positively skewed. The geochemical data were then log-transformed to obtain a log-normal distribution. In addition, the data set was checked for outliers using Tukey boxplots [70] and the resultant data subset was used in threshold calculation as follows:

$$Threshold = median + 2MAD$$

Multivariate statistical analyses (correlation matrix and factor analysis) were then applied to explore and investigate the data structure, decipher trends and relationship between variables; and infer the underlying factors influencing the stream sediment geochemistry.

#### Map production

Coloured geochemical maps of the data subset were drawn using the ESRI ArcMap 10.2 software package. For interpolation in a grid format, the inverse distance weight (IDW) technique was employed. A maximum of 15 neighboring samples was used for the estimation of each grid point and a power of 2 was chosen to achieve some degree of smoothing. The geochemical data were then classified based on the percentiles 5, 25, 50, 75, 90 and 98% and colour-coded according to this range. Highest concentrations were shown in hot colours while the lowest ranges were shown in cold colours. Also, graduated symbol plots of factor scores of the element associations obtained by factor analysis were produced to examine their relationship with the basin geology.

## Results and discussion

### Mineralogical composition

XRD analysis identified the following mineral phases: ubiquitous quartz (39–86%), moderate amounts of phyllosilicates (micas + clay minerals) and feldspars (Table 1). Rutile and gismondine were identified in only two samples (Table 1). This variability in mineralogy reflects the composition of the complex basement geology dominated by migmatitic gneisses, granites, metasedimentary and metavolcanic rocks (Fig. 2). The predominance of quartz in the sediments is likely due to reworking of sediments along flow paths. In a typical tropical basin like the study area, the high intensity of chemical weathering may also contribute to the modification of the sediment composition [71]. Additionally, hydraulic energy and sorting are also known to influence the mineral composition of sediments [72]. The samples GB15, GB20 and GB44 were collected in streams where the water flux was low. Thus, this accounts for the moderate phyllosilicates content in the bottom sediments.

**Table 1:** Semi-quantitative mineralogical composition of selected stream sediments and representative rock types of the lower Lom Basin

	GB15	GB20	GB24	GB34	GB44	GB47	Range
Quartz	82	39	79	86	82	72	39–86
Phyllosilicates	45	32	0	0	10	0	0–45
Feldspars	0	12	12	0	0	25	0–25
Rutile	0	27	30	0	0	0	0–30
Gismondine	0	25	0	27	0	0	0–27
Rock type	Upper gneiss	Embrechyte	Granodiorite	Lower gneiss	Schists, quartzite, conglomerates	Granite	

*GB* Garoua-Boulai Betare-Oya

### Trace metal content and sediment quality assessment

Descriptive statistics of trace metal concentrations in stream sediments are presented in Table 2. Pronounced deviations between means, medians, standard deviations and MADs were observed. Also, all the selected metals were positively skewed implying the influence of extreme values, the presence of multiple populations and the effects of analytical precision or limits of detection of the data set [73]. The mean value of pH = 6.4 indicates near neutral conditions of the catchment. Elemental composition showed a wide variation which is likely generated by the physical and chemical weathering processes operating within the drainage basin [74, 75]. From the analytical results, Cd (2.65 µg/kg) had the lowest mean concentration followed by Hg (5.40 µg/kg), Se (48.55 µg/kg), Co (92.85 µg/kg) and As (99.40 µg/kg) (Table 2). These elements are usually present in trace amounts in rocks as reported in the mean background contents in the continental crust [76]. Iron and Mn had the highest mean concentrations of 28.325 and 442 mg/kg, respectively. When compared to regional stream sediment surveys in other Sub-Saharan Africa regions [26, 77], the Lom Basin sediments were depleted in all examined trace metals. However, average concentrations of the non-essential trace metals As (99.40 µg/kg), Hg (5.40 µg/kg), V (963.14 µg/kg) and Pb (151.59 µg/kg) were significantly higher than the levels (As = 22.3 µg/kg, Hg ≤ 0.01 µg/kg, V = 158.5 µg/kg and Pb = 12.3 µg/kg) reported by Taiwo and Awomeso [37] for sediment from the gold city of Ijeshaland. Also, all the trace metals analyzed showed a similar fingerprint in the stream waters of the study area characterized by low levels (< 1 µg/L) of V, Cr, Co, Cu, Zn, Cd, Pb and significant concentrations of Fe (20–5011 µg/L) and Mn (0.2–248 µg/L) [53]. Possible reasons for the depletion of these trace metals may be a reflection of the impoverished bedrock and the neutral pH which does not favour the dissolution and mobilization of the trace metals from the sulphide gold-quartz veins [78].

**Table 2:** Summary characteristics of stream sediment geochemical data and Se:Hg ratios in the lower Lom Basin (N = 55)

Element	Unit	Minimum	Median	Mean	Maximum	MAD	Std Dev	Variance	CV %	Skewness
Sc	µg/kg	2.11	129.97	211.75	1699.18	4.22	328.78	108,095.73	155.27	3.11
V	µg/kg	53.57	85.96	963.14	12,333.20	5.20	2365.99	5,597,892.54	245.65	3.28
Cr	µg/kg	13.58	39.18	763.93	10,052.57	1.26	1941.97	3,771,266.78	254.21	3.43
Mn	mg/kg	224.12	319.00	441.56	1441.56	5.86	273.94	75,040.66	62.04	2.10
Fe	mg/kg	12,109.19	22,678.94	28,325.12	159,319.95	6985.00	22,219.08	493,687,443.27	78.44	4.29
Co	µg/kg	0.03	0.47	92.85	1308.38	0.14	256.17	65,625.13	275.90	3.41
Ni	µg/kg	5.59	15.79	239.02	3125.54	0.19	607.61	369,194.94	254.21	3.35
Cu	µg/kg	12.19	211.18	362.64	3946.64	0.04	680.27	462,768.04	187.59	3.95
Zn	µg/kg	2.61	315.39	573.24	4657.72	18.39	1008.88	1,017,834.67	176.00	3.11
As	µg/kg	0.35	22.66	99.40	1178.11	20.05	249.26	62,129.44	250.75	3.21
Se	µg/kg	0.10	21.03	48.55	509.56	3.82	100.73	10,145.84	207.47	3.23
Cd	µg/kg	1.30	2.48	2.65	7.85	5.37	0.98	0.97	37.10	3.69
Hg	µg/kg	0.19	2.35	5.40	83.24	18.26	11.87	140.85	219.70	5.55
Pb	µg/kg	7.25	47.64	151.59	2215.78	2168.13	365.78	133,795.98	241.30	4.10
pH	–	3.60	6.40	6.38	7.10	–	0.51	0.25	7.99	− 3.17
Se:Hg	–	0.04	2.70	2.31	7.49	–	–	–	–	–

*N* number of sampling sites, *MAD* median absolute deviation, *Std Dev* standard deviation, *CV* coefficient of variation

Table 3 shows the background, mean and threshold values of the trace metals in comparison with analytical results from other studies in order to obtain a preliminary inspection of the level of contamination in the bottom sediments. The median was chosen in this study as the local background because it is representative of the local data and less affected by outliers [79]. Threshold values were computed for the geochemical data subset following the elimination of outliers. The statistically derived threshold values are crucial in distinguishing between geogenic and anthropogenic sources of the trace metals [80]. These values represent the upper limit of the background concentrations of the potential toxic trace metals in sediments [10] and allows the identification of anomalous concentrations. Because stream sediments are an essential and dynamic component of catchments, they reflect the average chemical composition of the mixture of soils, sediments and rocks [32]. In this regard, the trace metal contents in this study were presented alongside local background levels in soils [49] and mean concentrations in ferralsols of the Sub-Saharan Africa region [75]. All trace metal concentrations (with corresponding data in local soils) in the Lom sediments were lower than the mean levels in local soils. About 18% of the sediment samples exceeded the concentrations of Fe and Mn in ferralsols. Based on this comparison, low concentration of trace metals in the sediment is likely due to the interplay of deep weathering, depleted parent rocks and the incorporation of metals in ferruginous clays or organic matter in the lateritic soil cover.

**Table 3:** Geochemical background and threshold values (µg/kg) of stream sediments from the lower Lom Basin alongside local soil and Sub-Saharan ferralsols

	Sc	V	Cr	Mn (mg/kg)	Fe (mg/kg)	Co	Ni	Cu	Zn	As	Se	Cd	Hg	Pb
This study, background	128.3	85.9	39.1	319.0	22,627.4	0.5	15.7	211.1	314.3	22.1	15.3	2.5	2.3	47.5
This study, mean	105.4	201.4	156.1	406.1	25,899.3	12.3	58.4	174.8	248.7	24.1	19.7	2.4	2.9	56.5
Threshold values	132.4	96.0	41.4	330.7	36,692.6	0.7	15.8	211.2	354.3	61.2	34.4	7.4	39.0	142.6
Local soil (ppm)	–	–	–	–	–	–	–	1	4	0.05	–	1	–	5
Ferralsols (mg/kg)	–	30	53	578	34,957	–	21	21	41	–	–	–	–	36
Percentage (%) sediment samples exceeding ferrasols	–	0	0	18.18	18.18	–	0	0	0	–	–	–	–	0

Geochemical background: median; Geochemical background threshold: median + 2 (median absolute deviation); metal concentrations in local soil are adapted from Manga et al. [49] while data for ferralsols are after Towett et al. [75]

The degree of correlation between trace metals in the bottom sediments is given in Table 4. Interestingly, except for Fe and Mn, all trace metals correlated negatively with pH. The poor correlations suggest these metals are relatively immobile at near neutral pH, thus accounting for their low concentrations. Similarly, Fe and Mn correlated poorly with all other elements suggesting a less co-precipitation effect on them in this drainage system. Unlike Fe and Mn, strong positive correlations were observed between Co–Cr–Ni–As–Se–V–Pb and V–Cr–Co–Ni–As and likely indicate that they are sourced from the granitic rocks and partial dissolution of sulphides within the catchment. Indeed, the complete dissolution of metal sulphides is effective under acidic conditions rather than the near neutral pH (mean = 6.4, Table 2) of the sediments.

**Table 4:** Correlation matrix of trace metals in stream sediments from the lower Lom basin at p < 0.05 (N = 55)

	Sc	V	Cr	Mn	Fe	Co	Ni	Cu	Zn	As	Se	Cd	Hg	Pb	pH
Sc	1.00														
V	0.28	1.00													
Cr	0.19	0.98	1.00												
Mn	0.12	0.09	0.09	1.00											
Fe	− 0.05	− 0.02	− 0.02	0.69	1.00										
Co	0.08	0.91	0.94	0.06	− 0.03	1.00									
Ni	0.20	0.98	0.99	0.09	− 0.02	0.93	1.00								
Cu	0.76	0.34	0.24	− 0.08	− 0.18	0.10	0.26	1.00							
Zn	0.70	0.32	0.23	− 0.05	− 0.21	0.08	0.26	0.96	1.00						
As	0.33	0.79	0.74	0.04	− 0.07	0.68	0.76	0.40	0.38	1.00					
Se	0.30	0.71	0.70	0.22	0.14	0.65	0.70	0.32	0.32	0.35	1.00				
Cd	0.52	0.33	0.27	0.15	− 0.05	0.25	0.27	0.38	0.41	0.44	0.16	1.00			
Hg	0.81	0.21	0.10	0.21	0.07	0.02	0.13	0.77	0.71	0.27	0.37	0.42	1.00		
Pb	0.65	0.65	0.54	− 0.08	− 0.16	0.39	0.57	0.85	0.82	0.63	0.51	0.47	0.67	1.00	
pH	− 0.22	− 0.46	− 0.46	0.08	0.06	− 0.41	− 0.50	− 0.31	− 0.34	− 0.40	− 0.48	− 0.18	− 0.18	− 0.42	1.00

*N* number of sampling sites

**Fig. 3:**
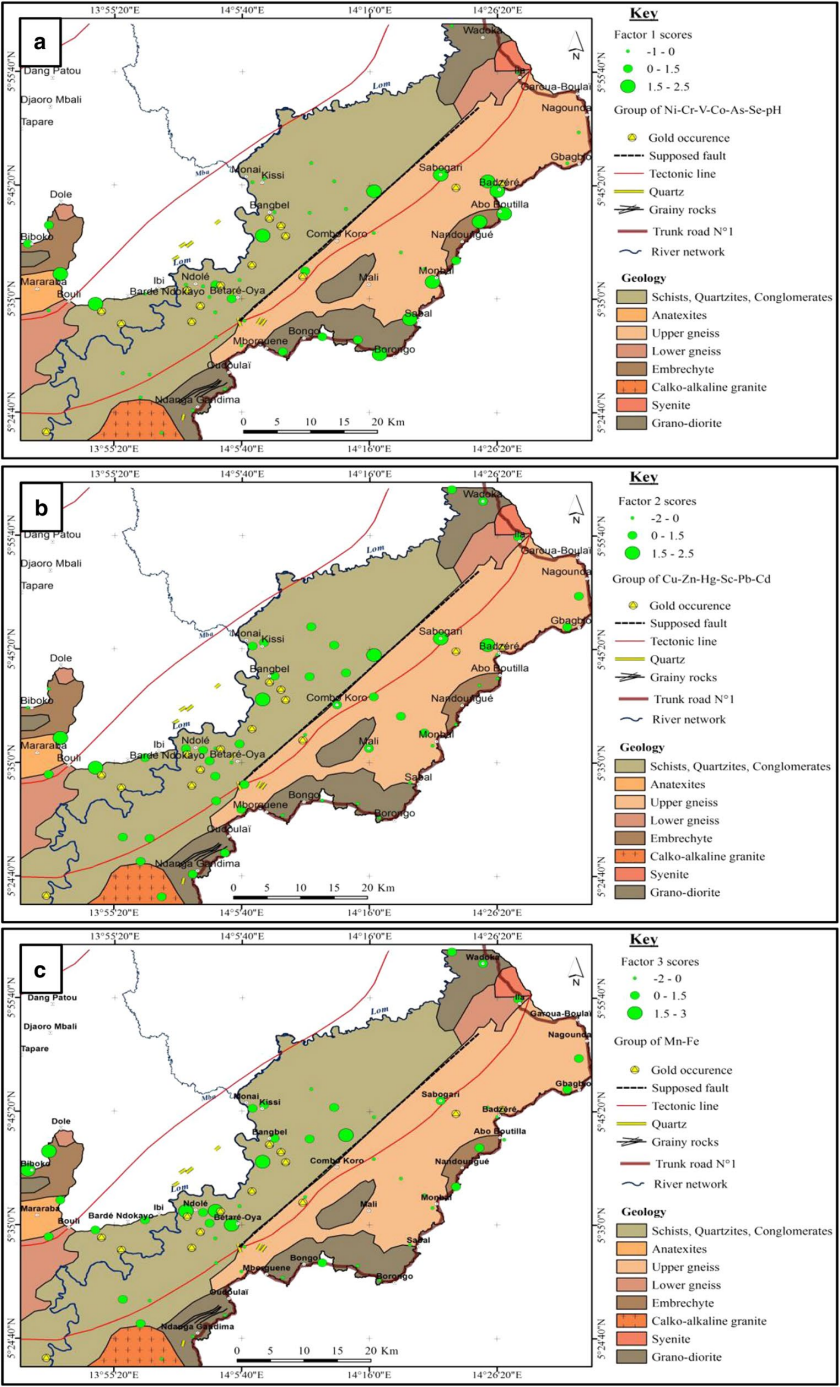
Spatial distribution of factor scores for **a** F1 association, **b** F2 association and **c** F3 association, in relation to geology. In each graduated symbol plot, the elements are ordered in decreasing loading

Factor analysis was applied on the log-transformed geochemical data to infer the controlling factors behind multi-element associations in relation to catchment geology, mineralization or anthropogenic activities. The spatial distribution of the factor scores of the element associations and their relationship with the geology are shown in Fig. 3a–c. Three factors explaining about 79% of the variance were generated (Table 5).

**Table 5:** Factor analysis with varimax rotation for 14 trace metals in stream sediments from the lower Lom Basin (N = 55)

Variable	Factor 1	Factor 2	Factor 3	Communality
Ni	*0.983*	0.117	0.018	0.981
Cr	*0.982*	0.091	0.022	0.974
V	*0.965*	0.206	0.021	0.974
Co	*0.958*	− 0.041	0.019	0.920
As	*0.749*	0.319	− 0.064	0.668
Se	*0.705*	0.269	0.240	0.627
pH	*− 0.529*	− 0.244	0.138	0.358
Cu	0.154	*0.928*	− 0.173	0.914
Zn	0.151	*0.904*	− 0.178	0.871
Hg	0.029	*0.897*	0.205	0.846
Sc	0.095	*0.879*	0.080	0.788
Pb	0.487	*0.790*	− 0.162	0.888
Cd	0.245	*0.537*	0.092	0.357
Mn	0.060	0.066	*0.919*	0.853
Fe	− 0.025	− 0.085	*0.892*	0.803
Variance	5.477	4.478	1.868	11.823
% Variance	36.515	29.851	12.451	78.817

The geochemical data was log transformed prior to factor analysis. Three factors were extracted

Factor loadings ≥ ± 0.500 are in italics

*N* number of sampling sites

Factor 1 accounts for 36.5% of the total variability. This dipolar factor showed high positive loadings for Ni, Cr, V, Co, As, Se and a negative loading for pH. The F1 association showed high and medium factors scores relating to the metamorphic basement of the catchment (Fig. 3a). Sediments derived from granitic rocks and other felsic metasedimentary rocks such as quartzites and amphibolitic schists that make up the Lom basement are known to be poor in V, Cr, Ni, Co, and As [79.] Besides, Co, V and Ni can easily replace Fe in magnetite [81] which is a major oxide in the ferralitic soils of this tropical basin [82]. The presence of As in this factor is attributable to arsenopyrite dissemination in the parent rocks. Arsenopyrite has been reported as a separate sulphide mineralization event distinct from the main chalcopyrite sulphidation in the study area [66]. The negative contribution of pH in this factor implies that an acidic environment is required for these metals to be released from their geological materials.

A significant proportion of data variability (29.9%) described by Factor 2 is associated with scores of chalcophiles (Cu–Zn–Hg–Pb–Cd) and Sc. High factor scores (> 0) of these elements occur around reported gold indications reflecting sulphide gold-quartz vein mineralization (Fig. 3b). Moreover, previous studies have reported the occurrence of chalcopyrite (Cu), sphalerite (Zn) and galena (Pb) in the underlying rocks of the study area [45, 83]. The negative association of As and chalcophile elements is consistent with the claims that two distinct hydrothermal events are related to the epithermal gold mineralization in the study area [66]. Also, As and Pb have been identified as potential pathfinder elements for gold in the area. The contents and geochemical dispersion haloes of these metals in different lateritic profiles in the area were used to indicate gold mineralization. Arsenic was widely dispersed in soils and considered useful in regional survey while Pb was suited to follow up work [82]. Scandium is likely associated with organic matter. Its small size and high charge favour the formation of stable organic complexes in soils or adsorption on clay minerals derived from the chemical weathering of the granitic rocks [84].

The manganiferous relationship in factor 3 is a clear indication of the presence of Fe-bearing rocks and the co-precipitation effect. Accordingly, the highest F3 factor scores are located in the area underlain by the volcaniclastic schists and the metasedimentary rocks, quartzites and metaconglomerates (Fig. 3c). Iron and Mn exist as compensating ions on clay complexes and their precipitation is mainly dependent on the pH of the sediments in the catchment [85]. Hence, the poor correlation observed between Fe and Mn and the other trace metals suggests that they do not play a major role in scavenging these elements under near neutral conditions.

### Spatial geochemical features

**Fig. 4:**
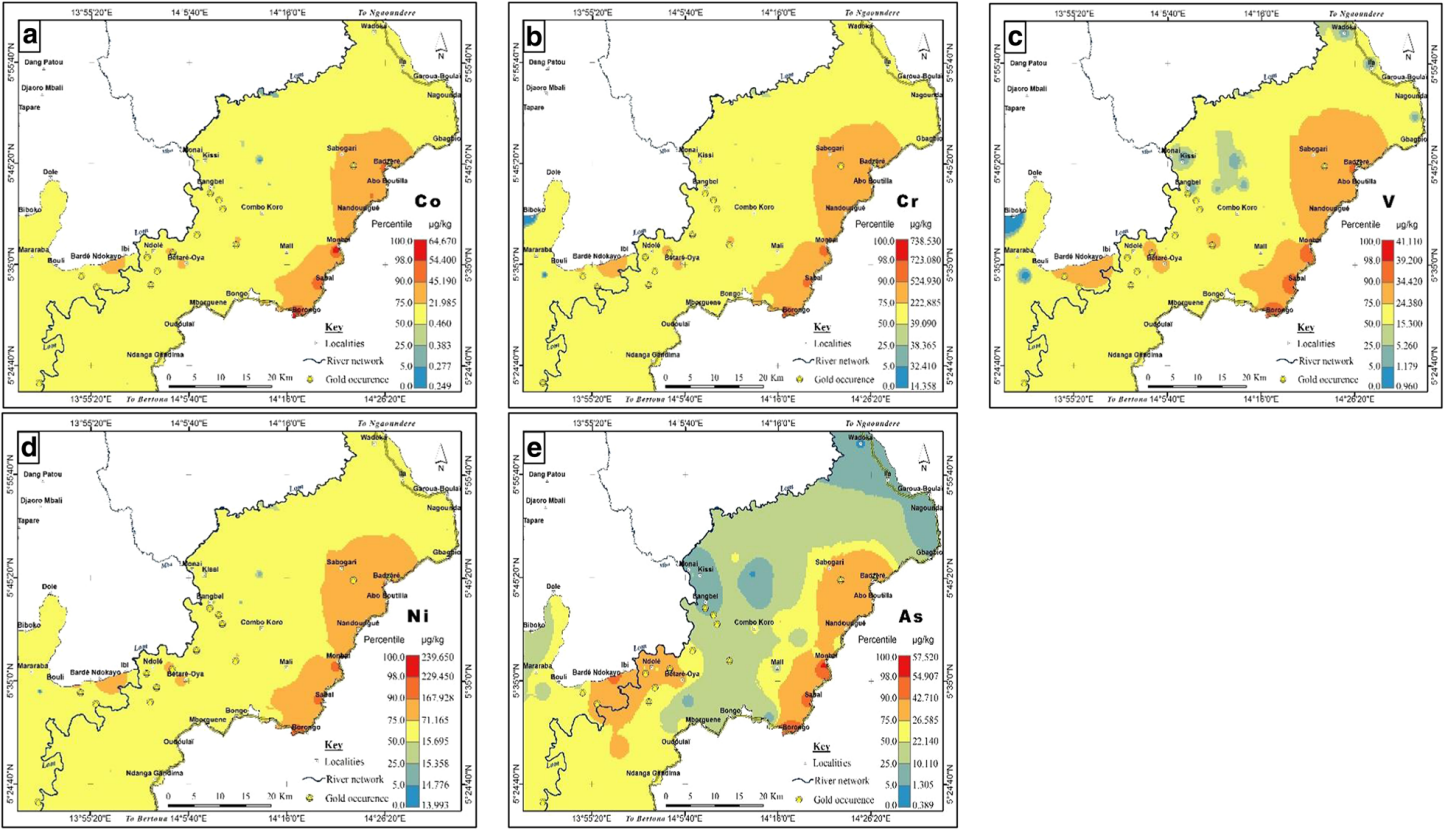
Geochemical background of **a** Co, **b** Cr, **c** V, **d** Ni and **e** As in the lower Lom Basin Co, Cr, V and Ni were slightly enriched in relation to the estimated threshold values

Spatial distributions of high levels of Co, Cr, V and Ni (Fig. 4a–d) cluster in the eastern part and correspond to areas underlain by upper gneisses and granodiorite (Fig. 2). Similarly, As distribution (Fig. 4e) is controlled by the catchment geology even though its concentrations were lower than the calculated threshold (Table 3). Moreover, relatively high concentrations of As coincided with some reported gold occurrences in the area. As previously stated, this observation is in line with the assertion that As is an important pathfinder for gold in this basin [82].

**Fig. 5:**
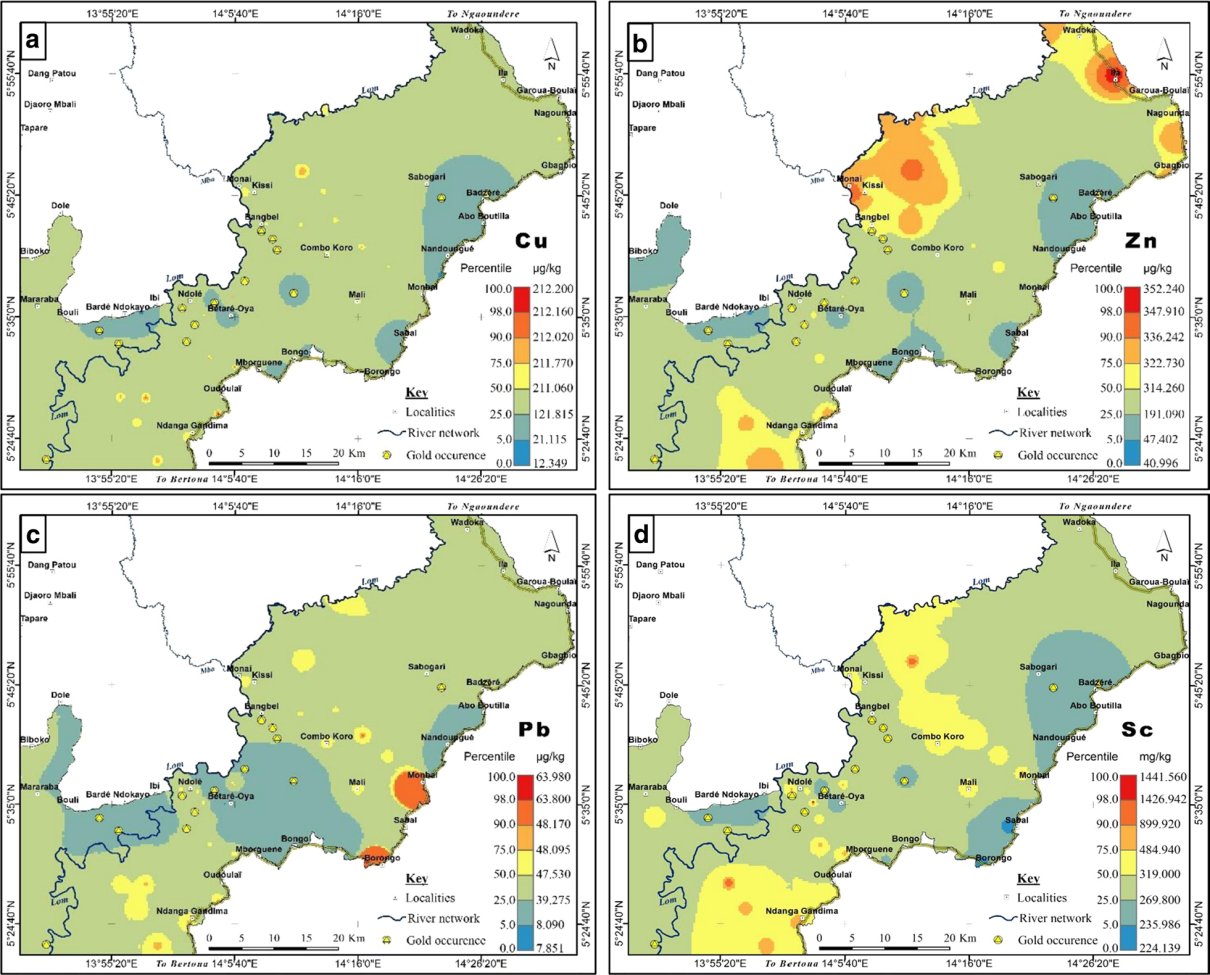
Geochemical background of **a** Cu, **b** Zn, **c** Pb, and **d** Sc in the lower Lom Basin

Contrary to As, the base metals Cu, Zn, Pb and Sc have very low values around gold deposits (Fig. 5a–d; for gold occurrence, see Fig. 2). No anomalous sites were observed for these metals and their background concentrations indicate sulphide mineralization related to vein gold deposits. Whole rock geochemistry of the gold-quartz veins by Vishiti et al. [48] suggests a generally low base metal content resulting from the reaction between the hydrothermal fluid and the granitic rocks in the area. This explains the very low concentration of the base metals in the bottom sediments.

**Fig. 6:**
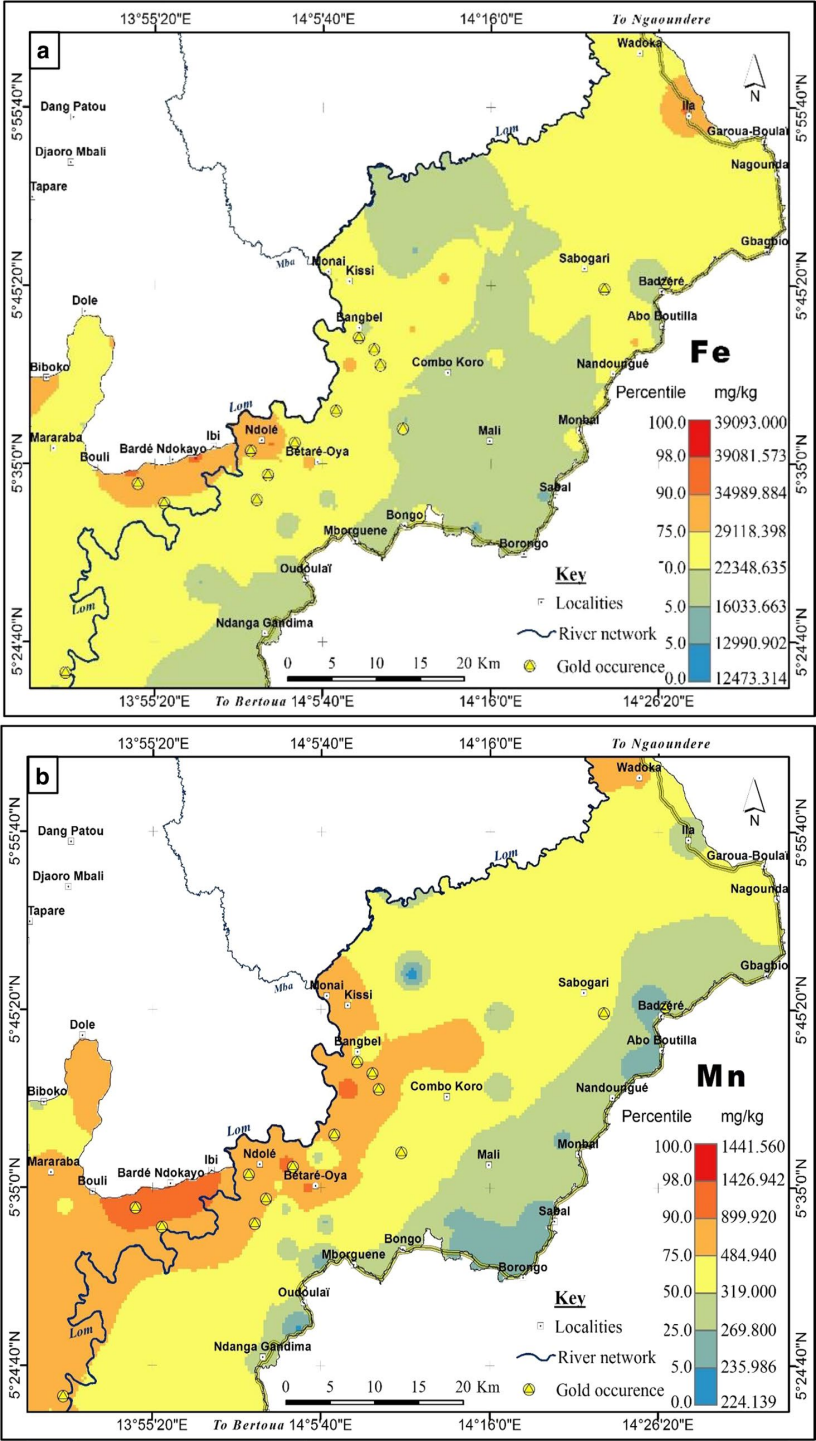
Geochemical background of **a** Fe, and **b** Mn in the lower Lom Basin

Hot spots of Fe and Mn occur in the NW-SW portion of the study area (Fig. 6a, b). These elements are important constituents in ferromagnesian silicates and oxides in the underlying metamorphic rocks. In addition to the hypogene and supergene hematite sources, other possible sources of Fe in the studied sediments are the sulphide minerals pyrite and arsenopyrite associated with the primary gold bearing quartz veins [48]. Besides Cu, As and Pb, Fe is considered as another pathfinder for gold in the area [46]. Hence, deep chemical weathering in this tropical basin results in the enrichment of Fe and Mn in the sediments. In the light of environmental significance, Fe and Mn are environmental scavengers. Heavy metals such as Cu, Zn and Pb and the metalloid As can form stable complexes with Fe and Mn oxides through adsorption or co-precipitation processes [86]. Also, Fe and Mn oxides form thin coatings on minerals and clay particles which serve as natural traps or carriers of the heavy metals discharged into the aquatic system [87].

**Fig. 7:**
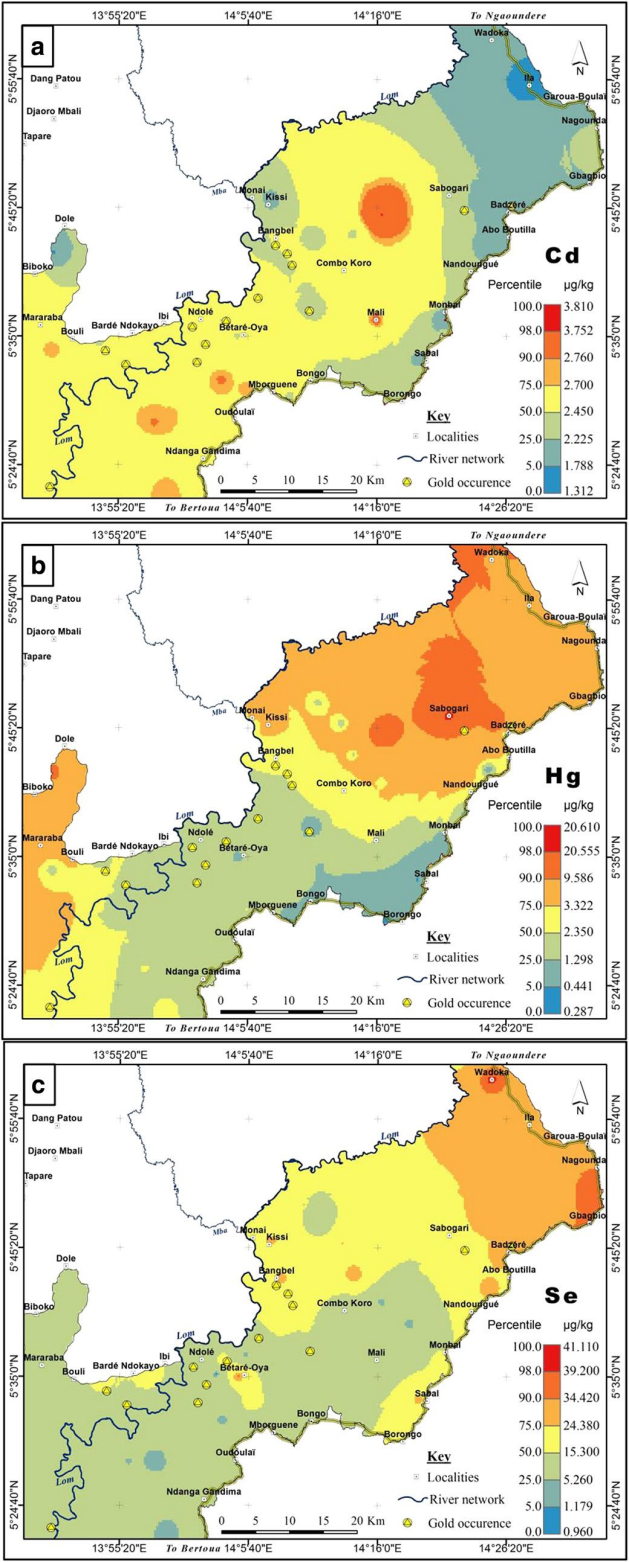
Geochemical background of **a** Cd, **b** Hg and **c** Se in the lower Lom Basin

The distribution patterns of Cd, Hg and Se were distinct (Fig. 7a–c). Cadmium had lower concentrations (maximum concentration = 3.8 µg/kg) compared to the estimated threshold (13.21 µg/kg) and crustal average (98 ppb) [88]. Despite the dissimilar distribution trends for Se and Hg (Fig. 7b, c), Se:Hg ratios can be used to check for Hg contamination in sediments [89]. The Hg-to-Se molar ratio was first proposed by Ganther et al. [90] as a reference standard for Hg contamination. Ralston [91] later suggested that Se:Hg > 1 implies Se plays a key role in Hg assimilation processes. Using this approach, the ratios were calculated and presented in Table 2. Molar ratios above 1 indicate low Hg content and a protective effect of Hg toxicity, and vice versa. The estimated ratios ranged from 0.04 to 7.49. More than 80% of the samples had Se:Hg > 1 indicating that the sediments had higher Se contents over Hg. Thus, the negative effects of Hg are neutralized by the relatively higher Se content through assimilation processes. In the study area, gold amalgamation is practiced [92] and therefore, a plausible source of Hg in the sediments. Naturally, Hg occurs in trace amounts in the earth’s crust [93]. Through its use in mining, Hg may be discharged into water, deposited in sediments or released into the atmosphere. Mercury is a toxic substance which affects the reproductive and nervous system [94]. It is therefore important to monitor the use of the hazardous element in mining within the catchment (Fig. 7).

## Conclusions

For the first time, the mineral composition, background values, threshold values and baseline environmental geochemical assessment of stream sediments from the lower Lom Basin have been made available. Mineralogically, quartz, phyllosilicates (muscovite + kaolinite) and feldspars constitute the dominant mineral phases in the sediments. These minerals are derived primarily from the weathering of the complex plutono-metamorphic basement and influenced by hydraulic energy and sorting. In terms of trace metals, concentrations of Sc, Cu, Zn, As, Se, Cd, Hg and Pb were low while V, Cr, Co, Ni, Mn and Fe were slightly enriched compared to their calculated threshold values. Overall, the low trace metal content of stream sediments is the result of the interaction of the near neutral pH of sediments (which does not favour the dissolution of metal sulphides), impoverished bedrocks and chemical weathering.

Multivariate statistical techniques enabled us to comprehend the basic processes influencing spatial geochemical variability. The spatial distribution of the trace metals Ni, Cr, V, Co and Se is controlled largely by source geology. Arsenic distribution showed a coherent relationship to the occurrence of Au deposits in some parts of the study area. Mercury, a hazardous environmental pollutant, is released into the basin through its use in gold recovery. Its continued use in refining gold may lead to harmful levels in the sediments.

The results obtained from this study show that the sediments have not been impacted by mining practices. However, given the paucity in fundamental geochemical data in Cameroon, this newly generated stream sediment data will serve as guidelines for future studies (environment, health and agriculture) in the region and other mineralized areas in the country. Future work should include the examination of metal composition in environmental samples from abandoned and active mine sites for comparison and environmental health risk assessment.
